# The dynamics in food selection stemming from price awareness and perceived income adequacy: a cross-sectional study using 1-year loyalty card data

**DOI:** 10.1016/j.ajcnut.2024.03.003

**Published:** 2024-03-07

**Authors:** Mikael Fogelholm, Henna Vepsäläinen, Jelena Meinilä, Cameron McRae, Hannu Saarijärvi, Maijaliisa Erkkola, Jaakko Nevalainen

**Affiliations:** 1 Department of Food and Nutrition, University of Helsinki, Helsinki, Finland; 2 Desautels Faculty of Management, McGill University, Montreal, Canada; 3 McGill Centre for the Convergence of Health and Economics, McGill University, Montreal, Canada; 4 Faculty of Management and Business, Tampere University, Tampere, Finland; 5 Faculty of Social Sciences, Unit of Health Sciences, Tampere University, Tampere, Finland

**Keywords:** food price, food purchase, socioeconomic status, nutritional profile, grocery store, retail

## Abstract

**Background:**

Higher cost of healthy foods may explain unhealthy dietary patterns in lower-income households. Unfortunately, combining food selection and nutrient intake data to price and expenditure is challenging. Food retailer’s customer loyalty card data, linked to nutrient composition database, is a novel method for simultaneous exploration of food purchases, price, and nutrition.

**Objectives:**

We studied the associations between perceived income adequacy (PIA) as a grouping variable with price (per kilogram or megajoule) and the volume of purchases (percentage of expenditure or energy) simultaneously as outcome variables for 17 most purchased food groups.

**Methods:**

We used 1-year (2018) loyalty card data from the largest grocery chain in Finland. Participants were 28,783 loyalty cardholders who made ≥41% of food purchases from the retailer and answered an online questionnaire at the midpoint of data collection. The 5-level PIA described the perceived financial situation in the household. Energy and nutrient content of foods purchased were from the Finnish Food Composition Database Fineli. We calculated the Nutrient Rich Food Index per 100 g food using 11 nutrients. Trends in prices and expenditures between PIA levels were analyzed using 2-sided Jonckheere–Terpstra tests, with false discovery rate control (Benjamini–Hochberg method) and confounder adjustments (inverse probability weighting).

**Results:**

Lower PIA participants selected cheaper foods per kilogram and megajoule within most food groups. They also favored unhealthy food groups cheap in energy [<1 € (USD 1.18)/MJ]. Despite lower purchase price, the expenditure (%) among lower PIA was higher on alcohol, snacks, sugar-sweetened beverages, and sweets and chocolates.

**Conclusions:**

Participants with lower PIA showed stronger price awareness. It is crucial to consider the pricing of competing alternative food groups, when steering toward environmentally sustainable and healthier food purchases. Package labeling might also direct the selection of healthier choices among the less expensive items within a food group.

## Introduction

A plant-dominant, mixed diet has been the base of dietary guidelines for decades [1,2]. Examples of these kinds of healthy dietary patterns are the traditional Mediterranean diet [3] and the “Healthy Nordic diet” [4]. More recently, the focus on environmental sustainability has emphasized the importance of decreasing the consumption of animal proteins and a concomitant increase in the use of legumes and other sources of plant protein [5]. However, dietary guidelines are not reached in any part of the world [6]. There are several reasons explaining unhealthy dietary habits, including both individual and environmental determinants: individual motives focusing more on convenience or taste than health [7,8], poor availability of healthy foods [9], and marketing of unhealthy food and confusing health claims [8,10,11].

In addition, higher cost of healthy foods and diets may explain unhealthy dietary patterns among individuals and households with lower income [8,[12], [13], [14]]. We have earlier found that cheapness as a motive for food selection was associated with both low educational attainment and income [7]. Foods with higher energy density often cost less per unit of energy compared with foods with higher nutrient density [12,15]. Therefore, diets with lower nutritional value and with a higher content of, for example, added sugars and saturated fats, are generally less expensive per unit of energy [16]. However, some studies have indicated that diets higher in plant-based protein sources need not cost more per unit of energy [13] or in total [17].

Grocery stores can be seen as a localized food environment where consumers interact with the food system [18]. Understanding the dynamics among food purchases, nutrient intake, price, and expenditure would significantly contribute to the design and implementation of food policies that foster the transformation toward a plant-based diet. However, combining data on food selection to price and expenditure is challenging [12]. This is because these data sets are usually collected, organized, and owned by organizations that have differing purposes and needs, such as retailers, health agencies, or national statistical institutes. Moreover, linking dietary assessment data with average retail prices does not usually include price variations (e.g., weekly, seasonal, discounts, and type of store) [12,16,19].

Our research group has obtained access to over 2 years of day-to-day food purchase data obtained from ∼47,000 loyalty cardholders of the largest grocery retail chain in Finland [20], combined with an extensive online questionnaire. In this study, our aim was to use these data to study the associations between perceived income adequacy (PIA) as a 5-level ordinal grouping variable and price (per kilogram or megajoule) and the volume of purchases (percentage of expenditure or energy) simultaneously as outcome variables for 17 food groups. The analyses provide novel insights into the socioeconomic determinants of food selection and their nutritional qualities. Our findings are of critical importance in understanding the role of price in food selection, especially for those with limited resources, and in steering everyone toward more sustainable food choices.

## Methods

### Study design and ethical review

Data were obtained from the S Group, which is the largest grocery retailer in Finland, with a market share of 46% in 2018 and a national coverage [21]. Members of the S Group’s loyalty card program have a card to be used when making purchases, and they receive ≤5% cash back for their purchases to their bank account. The retailer contacted the households’ primary cardholders across Finland through e-mail and invited them to participate in the study, which involved consenting to the release of their grocery purchase data for research purposes and voluntarily responding to an online questionnaire in summer 2018. Although it was possible to have >1 card in the household, we used data only from the main card that typically covers most food purchases (median 86% of household grocery purchases in S Group’s stores). The study was reviewed by the University of Helsinki Ethical Review Board in the humanities and social and behavioral sciences (Statement 21/2018). Each participant provided an informed consent electronically.

### Participants

In total, 47,066 loyalty cardholders consented to release their grocery purchase data. We did not have information on the number of valid e-mail addresses or the proportion of e-mails reaching the cardholders (e.g., bypassing trash e-mail filters). Of those who gave their consent, 36,621 (78%) responded to the online questionnaire including the PIA question. For inclusion, we set a threshold of self-estimated degree of loyalty at ≥41% (19% of the respondents had lower); the threshold value was based on an earlier observation that the relative proportions of purchases of main food groups were similar among these participants [20]. Moreover, we excluded participants whose annual purchases in 2018 were <100 € (3%). A total of 28,783 (79%) participants fulfilled these criteria and answered the key questionnaire items and, hence, were included in the analyses. The flow chart of the different stages in recruitment, inclusions, and exclusions is shown as Supplemental Figure 1.

### Background characteristics

Cardholders’ sex and age were obtained from the retailer’s database. We collected information on resources (education and household income) using the online questionnaire. Participants reported their education on a 4-point scale: primary school or less, upper secondary school, lower-level tertiary, or higher-level tertiary. Monthly gross household income was reported on a 7-point scale ranging from <1500 € to 9000 € or more. Scaled monthly household income was then calculated as the midpoint of the categories divided by the square root of reported household size (The Organisation for Economic Co-operation and Development (OECD) square root scale) [22].

The household PIA was the main explanatory variable in this analysis. Subjective income may reflect an individual’s economic utility better than that by objective (actual) income, particularly in sociodemographically diverse contexts [23]. Owing to high regional variation in costs of living and age-related indebtedness in Finland [24], we chose PIA instead of income. We queried PIA by the question: How would you describe the financial situation of your household and the amount of disposable money at the moment? The participants chose one of the given alternative answers: *1*) I/we must compromise on almost everything; *2*) I/we have to compromise on purchases from time to time; *3*) I/we manage when I/we purchase carefully; *4*) I/we get along quite nicely; *5*) I/we get along very well; *6*) I cannot/I do not want to say. The Spearman correlation between PIA and household scaled income was *r* = 0.58 and between PIA and crude household income was *r* = 0.47.

### Food purchase variables

The grocery food purchase data used in this study covered the year 2018, from 1 January to 31 December. Each purchase was linked to the cardholder and associated with an item description, time stamp, and quantity (ie, weight, volume, or number of packages), location of purchase and cost of the food(s). We had information on the food group level, made by the retailer. Because these food groupings are not aligned with health or environmental impacts, we have conducted an excessive regrouping by a group of professional nutritionists to better serve the purpose of our studies [25]. We classified the food groups in 4 classes. The broadest (crudest) level of hierarchy (class 1) includes 38 main food groups based on characteristics such as healthiness, environmental impact, and purpose of use **(**Supplemental Table 1). The sublevel classes are more detailed, and homogeneous regarding the abovementioned characteristics. Examples of the most detailed classification of foods (class 4) are sugar-sweetened beverages, low-fiber cereal, and wild fish. We refer to this class by the term “foods,” to separate them from the less detailed classes including “food groups.”

For this analysis, we aimed at a restricted number of food groups that would provide meaningful interpretation for the association among PIA, price, and characteristics of purchased foods. The broadest level of hierarchy (class 1) was taken as the starting point. We excluded food groups with only marginal purchases, which we defined as <1% of total expenditure or <0.2% of total energy content of purchases. One of the groups, including bread and bakery products, turned out to be much larger in percentage of expenditure (EX%) and percentage of energy (E%) than the others. Hence, we divided this group into 3: bread, bakery (including pizza), and grain-based food (e.g., pasta and rice). For the same reason, we divided total dairy into cheeses and (other) dairy products. The final number of food groups in our analyses was 17.

### Energy content and nutritional profile of the purchases

Next, the foods and food groups were linked to their nutrient content. We used the Finnish Food Composition Database Fineli, version 20 (www.fineli.fi) [26]. The database is constantly updated and maintained by the Finnish Institute for Health and Welfare. The purchase volume (in kilograms) of each food group was multiplied by the energy content per 1 kg of the group to obtain the absolute energy content of the purchases. The energy contents of all purchased food groups were summed to obtain the annual energy content of the total purchases.

We used nutritional profiling for a quantitative analysis of the nutrient density as a proxy for healthiness of purchases [25]. We calculated the Nutrient Rich Food Index (NRFI) for each food following principles of Drewnowski and Fulgoni [27]. NRFI was calculated per 100 g of product using 11 nutrients, of which 8 were regarded as positive (protein, fiber, polyunsaturated fatty acids, calcium, iron, vitamin D, vitamin C, and folate) and 3 as negative (saturated fatty acids, saccharose and salt) regarding anticipated health effects. Recommended values used for the 11 nutrients were from the Finnish Nutrition Recommendations, which were similar to the Nordic Nutrition Recommendations 2012 [28], except salt which being ≤5 g in the Finnish recommendations (≤6 g in the Nordic Nutrition Recommendations 2012). The calculation has been described in detail elsewhere [25]. Finally, NRFI for the food group per individual was obtained by summing up the NRFIs for the food items multiplied by the volume of the purchase.

### Statistical analyses

Total purchase prices for individuals were calculated by first adding up the expenditure (in euros), energy (megajoules), and volume (kilograms) of the purchased food items by group on each loyalty cardholder. One euro (€) is approximately US $1.18 (average conversion rate for 2018), and 1 MJ is equal to 239 kcal. The individuals’ purchase prices of the foods in different categories were then obtained by dividing the expenditure of the group by either energy or volume of the group. Similarly, the proportion of energy and the proportion of expenditure from each food group were computed as the ratio of group-specific energy and expenditure to the total energy and total expenditure across all groups, respectively. Individual-level quantities were then summarized as medians for PIA levels because their distributions were skew.

To test for a trend in the prices, expenditures, and NRFI (outcome variables) between levels of PIA (ordinal grouping factors), nonparametric methods were chosen owing to deviations from a normal distribution. We used a 2-sided Jonckheere–Terpstra test, with a control of the false discovery rate by the Benjamini–Hochberg method set at 5% level. To control the potential confounding factors, we used the inverse probability weighting (IPW) method of the Jonckheere–Terpstra test [29]. In practice, we estimated the probabilities of falling into one of the PIA levels by a multinomial model, including education and sex as explanatory variables, and the inverses of these probabilities were used as weights. The effect size was estimated by relating the intragroup variance of the medians across different levels of PIA to the variability of the medians across food groups and PIA levels. SAS, version 9.4, was used in the analysis. The correlation of price per kilogram and per megajoule between the food groups was examined by using group-based median price per megajoule and per kilogram and the Spearman correlation coefficients across groups.

In the figures, we showed the data without confounder adjustment because these indicate the food selection of actual participant groups instead of weighted yet artificial groups that would match the IPW-adjusted Jonckheere–Terpstra tests. We focused on price per kilogram (in contrast to price per megajoule) because this is the price consumers see, it is likely to be related to their food selection, and it is not associated with energy density of the food [15].

## Results

Table 1 summarizes the background characteristics of the 5 PIA levels. Higher age, male sex, higher income, and higher education were associated with better PIA.

**TABLE 1:** Descriptive statistics of the participant loyalty cardholders, by perceived income adequacy (PIA) levels

Level description	Levels of PIA					Total
	1 (lowest): have to compromise on almost everything	2: have to compromise on purchases from time to time	3: manage when purchase carefully	4: get along quite nicely	5 (highest): get along very well	
*N*	1494	3500	8949	11,307	3533	28,783
Female (%)	72.9	75.9	69	63.5	57.1	
Age (y), median (Q1, Q3)	43 (33, 54)	41 (32, 53)	46 (35, 59)	49 (37, 62)	54 (41, 65)	47 (36, 60)
Age group (%)						
18–29 y	17.5	18.1	14.2	10.2	6.6	12.3
30–44 y	37	40.5	33	31.2	26.2	32.6
45–59 y	31.9	27.1	28.4	29.3	31	29.1
60 y	13.7	14.3	24.4	39.3	36.2	26
Education (%)						
Primary school or less	13.1	8	7.3	4.9	3.6	6.3
Upper secondary school	54.5	50.1	43.6	31.8	19.2	37.3
Lower-level tertiary	23.2	30.5	32.3	34.7	30.6	32.4
Higher-level tertiary	9.1	11.4	16.7	28.6	46.6	24
Household income (€^a^ per mo, OECD adjusted to household size), median (Q1, Q3)	1006 (750, 1677)	1875 (1299, 2348)	2250 (1591, 3031)	3712 (2625, 4125)	4500 (3712, 5250)	2651 (1874, 3750)

Abbreviation: OECD, The Organisation for Economic Co-operation and Development.

a 1 € = US $1.18.

**FIGURE 1:**
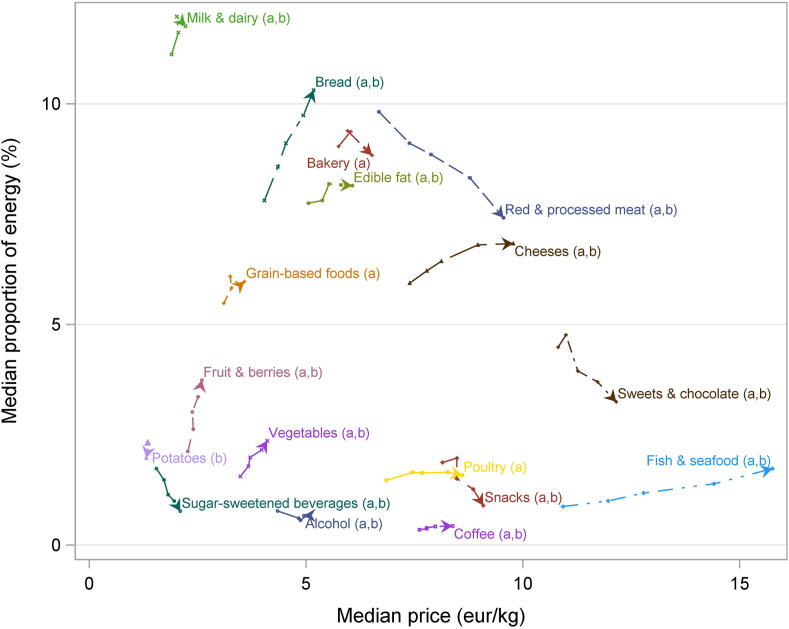
The association between perceived income adequacy (PIA) (5-level grouping variable) and median price (€/kg) and proportion of energy (%) as simultaneous outcome variables in different food groups. The arrow indicates to which direction the bivariate outcome changes after increase in PIA (1→5), except for alcohol beverages, in which PIA levels 4 and 5 are in a reverse order (indicated by the reverse arrow). Significant trend (*P* < 0.001), with Jonckheere–Terpstra test with false discovery rate correction and IPW, indicated by a being price per kilogram and b proportion of energy.

The median paid price (euros per kilogram) and the proportion of total energy of all purchases for 1 year (simultaneous outcome variables) for each food group are shown for the 5 PIA levels (ordinal grouping variable) in Figure 1. The results for the PIA levels are illustrated as dots, and the arrow shows the direction from the lowest to the highest PIA. Hence, an arrow pointing right indicates that higher PIA was associated with higher price per kilogram (or per megajoule). The slope shows the association between PIA and E% or EX%: a positive (increasing) slope indicates higher E% or EX% in those with higher PIA and a negative slope the opposite. The length of the line within 1 food group indicates the difference between PIA levels and shows the effect size: the longer the line, the larger the difference.

Excluding potatoes, PIA was positively associated with the price: within each food group, those with higher PIA purchased more expensive foods within that group (Figure 1, Table 2). The largest between-level variation in purchase price was observed for fish and seafood; red and processed meat; cheeses; poultry and poultry dishes; and sweets and chocolates, in a decreasing order of the effect size.

**TABLE 2:** Tests and quantification of differences in proportions of energy and expenditure purchased, and prices paid, between the 5 PIA levels (*N* = 28,783)

Food group	% Energy			% Expenditure			Price (€/kg)			Price (€/MJ)		
	Z^a^	*P*^a^	ES	Z^a^	*P*^a^	ES	Z^a^	*P*^a^	ES	Z^a^	*P*^a^	ES
Vegetables and fruits												
Vegetables	28.44	<0.0001	1.51	33.83	<0.0001	0.60	28.61	<0.0001	0.54	35.26	<0.0001	0.04
Fruits and berries	30.35	<0.0001	3.06	27.41	<0.0001	8.99	20.06	<0.0001	0.14	20.25	<0.0001	0.42
Cereal products												
Bread	26.67	<0.0001	7.39	26.49	<0.0001	3.44	44.13	<0.0001	1.80	44.88	<0.0001	0.19
Bakery	−0.10	0.923	0.46	−9.90	<0.0001	1.20	13.27	<0.0001	0.85	6.25	<0.0001	0.03
Grain-based foods	−0.30	0.762	0.41	−3.06	0.002	0.07	14.47	<0.0001	0.23	12.84	<0.0001	0.02
Edible fats	6.15	<0.0001	0.01	11.69	<0.0001	0.13	35.76	<0.0001	1.32	39.44	<0.0001	1.19
Fish and meat												
Fish and seafood	32.38	<0.0001	0.89	41.52	<0.0001	11.40	50.45	<0.0001	32.33	48.51	<0.0001	9.25
Red and processed meat	−12.97	<0.0001	6.26	10.77	<0.0001	1.37	63.63	<0.0001	11.19	62.10	<0.0001	3.34
Poultry	3.15	0.002	0.05	8.69	<0.0001	0.62	38.25	<0.0001	4.13	37.39	<0.0001	3.13
Dairy												
Milk and other liquid dairy	8.61	<0.0001	0.83	6.91	<0.0001	3.12	16.75	<0.0001	0.14	30.81	<0.0001	0.36
Cheeses	12.39	<0.0001	1.15	25.99	<0.0001	5.33	41.04	<0.0001	8.04	48.09	<0.0001	1.31
Discretionary foods and drinks												
Snacks	−29.10	<0.0001	1.51	−30.90	<0.0001	0.60	19.65	<0.0001	1.17	19.18	<0.0001	0.04
Sweets and chocolate	−17.34	<0.0001	2.85	−22.31	<0.0001	3.37	19.71	<0.0001	2.60	22.37	<0.0001	0.08
Sugar-sweetened beverages	−27.04	<0.0001	1.14	−24.93	<0.0001	1.49	28.29	<0.0001	0.39	29.51	<0.0001	1.30
Coffee	11.88	<0.0001	0.01	8.87	<0.0001	0.13	19.29	<0.0001	0.75	18.71	<0.0001	1.19
Alcohol	−4.34	<0.0001	0.05	−7.41	<0.0001	0.96	12.69	<0.0001	0.61	14.92	<0.0001	2.97

Nearly all tests reached statistical significance owing to the large size of the data, but they should not be interpreted as large differences without further interpretation. Effect sizes (ES), expressed in %, indicate how much variation within the food group (between PIA levels) was observed relative to the overall variation (between food groups and PIA levels). Large values indicate that the trend in the purchases of that food group had a strong gradient over the levels of PIA in the variable in question. For example, bread purchases in % energy varied markedly (∼7%) between PIA levels, but less so in price (0.19%, when considering € per megajoule).

Abbreviation: PIA, perceived income adequacy.

a Jonckheere–Terpstra test statistics and *P* values with inverse probability weighting adjustment for confounding. All statistically significant results remained so after false discovery rate correction too.

The association between PIA level and E% could be divided into 3 types of outcomes. In the first type, higher PIA was associated with higher E%: this was observed for bread, cheeses, coffee, edible fat, fish and seafood, fruit and berries, milk and dairy, and vegetables. The effect size for E% was >2 only for bread and fruit and berries. The second type included foods with a higher E% in those with lower PIA: alcohol, potatoes, red and processed meat, snacks, sugar-sweetened beverages, and sweets and chocolates. The effect size was >2 for red and processed meat and sweets and chocolates. Third, we found no association between PIA and E% for bakery, grain-based foods, and poultry. The unadjusted and IPW tests agreed to a large extent, with only a few minor changes in statistical significance.

**FIGURE 2:**
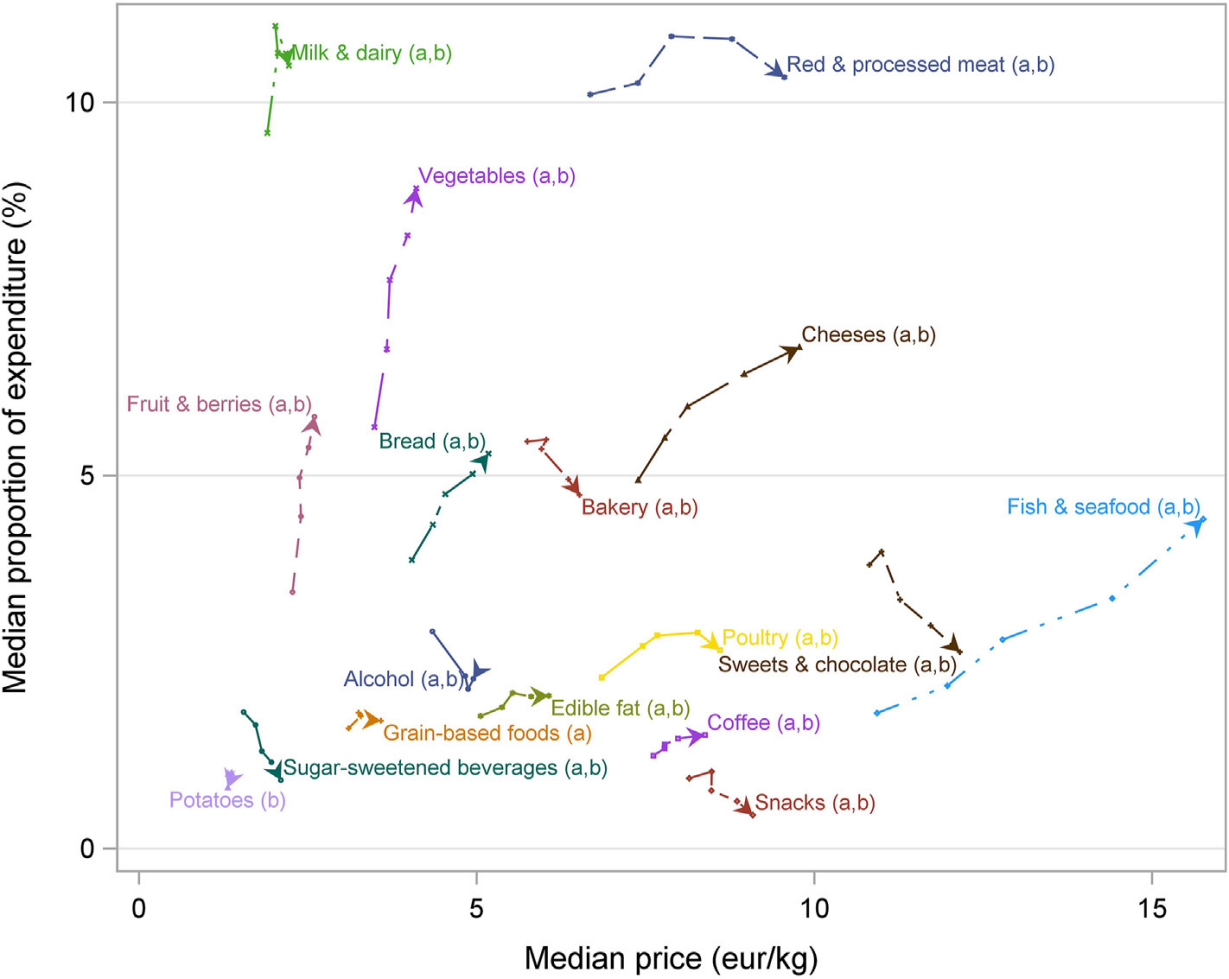
The association between perceived income adequacy (PIA) (5-level grouping variable) and median price (€/kg) and proportion of monetary expenditure (%) as simultaneous outcome variables in different food groups. The arrow indicates to which direction the bivariate outcome changes after increase in PIA (1→5), except for alcohol beverages, in which PIA levels 4 and 5 are in a reverse order (indicated by the reverse arrow). Significant trend (*P* < 0.001), with Jonckheere–Terpstra test with false discovery rate correction and IPW indicated by a being price per kilogram and b proportion of expenditure. IPW, inverse probability weighting.

The median paid price (euros per kilogram) and EX% of all purchases for 1 year for each food group are shown for the 5 PIA levels in Figure 2. Lower PIA was associated with higher EX% for alcohol, bakery, grain-based foods, potatoes, snacks, sugar-sweetened beverages, and sweets and chocolates, despite lower purchase price per kilogram (Table 2). The EX% on red and processed meat was similar in the lowest and highest PIA levels and slightly higher in PIA levels 3 and 4. By contrast, the proportion of expenditure was positively associated with PIA for food groups such as fruit and berries, fish and seafood, and vegetables. For fruit and berries and vegetables, the price variation (as indicated by effect size) was small, but the EX% results indicated clearly higher purchased volume (in kilograms) for the higher PIA levels.

Analyses including price (in euros) per food energy content (in megajoules) and E% by PIA levels are shown in Supplemental Figure 2. The results were similar to those using price per kilogram (Figure 1), indicating that there was a relationship between the price for kilograms and that for megajoules. Indeed, we saw a positive correlation within almost all 17 food groups (Supplemental Figure 3). By contrast, the results were different when price per kilogram and that per megajoule were compared between the food groups: the Spearman correlation coefficient across products for median price per megajoule and that per kilogram was not significant (*r* = 0.17; *P* = 0.12). Vegetables, alcoholic beverages, and coffee were the 3 most expensive food groups per energy [>2 € (USD 2.36)/MJ for median price], followed by fish and seafood, but the top 3 were not among the most expensive groups per kilogram (Supplemental Figure 3). Edible fats, grain-based foods, potatoes, snacks, bread, and bakery were the cheapest for the unit of energy [<0.5 € (USD 0.59)/MJ].

The association between PIA and the median purchased price per kilogram and nutritional profile (NRFI; a mathematical indicator for nutrient density as a proxy for healthiness) for the 17 food groups is shown in Supplemental Figure 4. Because the absolute values and differences of NRFI are somewhat difficult to interpret in a quantitative way, we focus our comments on the most apparent and visible trends within food groups. As indicated by an upward (positive) slope, the higher price paid by those with higher PIA was most clearly associated with better NRFI within the groups of fish and seafood, red and processed meat, and cheeses. The same positive association was also statistically significant for most other food groups, but the size of the differences in NRFI was small. Only poultry and poultry dishes and bakery had a statistically significant negative, but weak, association between the price paid by PIA levels and nutritional profile.

## Discussion

Recent developments in promotion of healthy and sustainable nutrition call for a food system approach and transformation [18]. It is also important to integrate social and economic perspectives with health and ecological sustainability. In this study, we used 1-year loyalty card data from almost 30,000 card holders to study the associations between PIA (grouping variable) and expenditure, sources of energy, price of purchases, and nutritional profile in 17 food groups.

The purchase patterns showed that individuals with higher PIA (those who felt they are doing economically well) chose more expensive items within nearly all food groups. This may be seen to allow more goods for limited resources [12,13]. As anticipated [30], nutrient-dense food groups, such as dairy, cheese, fruit and berries, vegetables, fish, and bread, constituted a larger proportion of energy (E%) and expenditure (EX%) in higher PIA levels. By contrast, discretionary foods, such as sugar-sweetened beverages, snacks, and sweets, had a larger E% and EX% in groups with lower PIA.

Recently, Love et al. [14] reported a positive association between income and consumption of fish but not for other protein sources (meat and poultry). The population with higher income chose more expensive fish and species with higher concentration of omega (ω)-3 (n–3) fatty acids. Although our results were similar, it should be noted that specifically ω-3 (n–3) fatty acids were not included in the NRFI equation we used. Nevertheless, the similar results obtained by different methods (NHANES data and prices from national-level sales projections) and population (the United States), compared with our data (loyalty card data from Finland), suggest that these findings are robust at least in high-income countries.

In contrast to fish, the association between price and nutrient density of purchases within other food groups was mostly weak. However, because we were operating with median NRFI values and price for each PIA level, there may still be single foods with a clearly healthier or unhealthier nutritional profile within a food group. Easy-to-interpret front-of-package labeling might direct people to select healthier choices, also among the less expensive items.

We interpret a positive relationship between price and purchase volume as an indication of preferred selection for higher PIA levels, whereas an opposite relation indicates foods preferred by lower PIA levels. Looking at the cheaper [<1 € (USD 1.18)/MJ] food groups, 4 (milk and dairy, bread, edible fats, and cheeses) had a positive and 6 (bakery, grain-based foods, sweets, potatoes, snacks, and sugar-sweetened beverages) a negative relation between price and purchases. Hence, by comparing the 17 food groups, we could not show that a low price per megajoule per se would be favored by those with lower PIA. Moreover, the cheapest food groups include both potentially healthy (e.g., milk and dairy, grain-based foods and bread, and edible fats) and unhealthy (e.g., red and processed meat, sweets, and sugar-sweetened beverages) foods.

Earlier data from France [31] suggested that food groups with more favorable nutrient profiles were higher in energy costs and that this might be a barrier to the adoption of dietary guidelines. We found that fruit and berries, poultry, fish and seafood, and vegetables were among the more expensive food groups (price >1 €/MJ). Two types of drinks, coffee and alcohol, also belonged to the most energy expensive foods. Regular coffee consumption has been associated with various health benefits [32], whereas excessive alcohol consumption is a health hazard [33]. Although we argue not all cheap foods were unhealthy, most energy expensive foods were healthy.

Lower PIA levels used more money in total for discretionary foods, despite choosing cheaper items. The question is whether the higher purchase volume is driving the low PIA level households to choose cheaper products within their favorite food groups. Our data do not support this because the lower PIA levels selected cheaper alternatives within all food groups, regardless of the relative amount of use.

Although the price per energy has been linked to socioeconomic differences in diets [13], it is not indicated directly to consumers—in contrast to the sticker price and price per kilogram. We observed similar associations when using price per energy or weight, against purchases, indicating that often a choice of lower price per weight leads to lower cost per energy. Our analyses do not tell whether the consumers compare prices not only within but also between food groups. The comparison might extend beyond a food group when groups are substitutes to each other, such as, meat, poultry and fish, and plant-based meat substitutes [34]. Given the lower price and better familiarity of meat, compared with plant-based alternatives, it is not surprising that most of the consumers stick to the traditional meat-dominant diet.

The loyalty card data used in this study is a novel and unique method to analyze the associations between socioeconomic status, food price, and purchase volume [20,34,35]. Our approach allows a longer follow-up period and a larger population, than more traditional methods to study dietary patterns and price [12]. Moreover, the method gives detailed assessment of actual purchases and prices. Variations of price by, for example, day, season, special offers, and store type and location are all included. Unlike other studies using loyalty card data [[36], [37], [38], [39]], we also obtained detailed background information on, for example, education, income, PIA, household size, and degree of loyalty.

Regarding weaknesses, our data set overrepresents females, smaller household sizes, high education, and high income, compared with the general Finnish adult population [21]. Only a part of total food purchases is from the retailer providing the data; however, we included cardholders who concentrate ≥41% of purchases to this grocery chain. Moreover, our data do not include eating out, which may affect the overall dietary quality if it is frequent. However, we compared the purchases with the cardholder’s personal diet, assessed by a short food-frequency questionnaire [35]. The correlation coefficients were in general of the same magnitude as when comparing a food diary and the frequency questionnaire [40,41].

We used nutrition profiling (NRFI) to give a more detailed picture on the nutrient density of purchases between and within the food groups [27]. This method is widely used, but limitations should be noted. There are many ways to calculate an index and the choice of equation and nutrients affect the outcome. In addition, a difference in NRFI is difficult to interpret in a quantitative way, particularly when comparing food groups. Our interpretation was that the nutrient density of the food basket primarily depended on the composition of different food groups in the basket, rather than on investing on more expensive food items within a food group.

Two retail chains dominate the Finnish food market, and we have data of the largest of these [22]. Both chains cover the whole country, and they are not strategically targeting any specific sociodemographic groups. Hence, we do not see that the choice of the retail chain would have caused any important bias in national representativeness.

As the main explanatory variable, we used PIA, not actual income. PIA is subjective and relative, and it indicates individual capacity to meet financial needs at present and in future [42]. Although actual income is positively associated with PIA, the association is modified by, for example, cost of housing, price of food, energy, and transportation. PIA may reflect an individual’s economic utility better than that by objective income [23]. In Finland, particularly the cost of living and transport varies in different parts of the country and types of communities (urban and rural). As a sensitivity analysis, we made the analyses shown in FIGURE 1, FIGURE 2 by using OECD-adjusted household income level, instead of PIA. The results (not shown) looked mostly similar, but they were, in general, more linear and clearer within many food groups when using PIA.

The cross-sectional study design prevented us from confirming a causal link between PIA and food selection based on price or nutritional quality. However, it is plausible that financial constraints drive the choice of cheaper options, as seen in previous research [7,8,12]. The use of a self-reported economic measure (PIA) could perhaps lead to reverse causality, if a limited food budget would influence participants’ perception of their income adequacy. In addition, unmeasured confounding variables could be present.

In conclusion, participants with lower PIA showed stronger price awareness. This was indicated by cheaper selection (per kilogram and per megajoule) within almost all food groups. Moreover, the food groups favored by lower PIA (ie, high proportion in expenditure and/or energy) were cheap in energy and classified as, according to nutrition recommendations, foods to be limited in the diet. By contrast, healthier food groups were favored by higher PIA participants, regardless of the price. Although not all cheap foods were unhealthy, it seems that most energy expensive foods were healthy. Easy-to-interpret front-of-package labeling might direct people to select healthier choices, also among the less expensive items. However, the nutrient density of the food basket primarily depended on the composition of different food groups in the basket, rather than on investing on more expensive food items within a single food group.

## Acknowledgments

We are grateful to the S Group (Finland) for sharing their customer loyalty card data.

## Author contributions

The authors’ responsibilities were as follows – MF, JN: designed research; JN: analyzed data; MF, JN: wrote the initial draft of the article, and all other authors contributed significantly to writing; MF, JN: had primary responsibility for final content; and all authors: read and approved the final manuscript.

### Conflict of interest

MF is a member of the S Group Advisory Board for Societal Responsibility. The membership is without any compensation. All other authors report no conflicts of interest.

### Funding

This study was supported by the Academy of Finland (grant numbers 350863, 2022 to JN and MF) and Social Sciences and Humanities Research Council (SSHRC), Canada (grant number 767-2021-2688, 2021 to CM).

### Data availability

Data described in the manuscript will not be made available because of contractual and privacy restrictions. Codebook and analytic code used for the manuscript are available on request from jaakko.nevalainen@tuni.fi.
