# Supplementary material for: The dynamics in food selection stemming from price awareness and perceived income adequacy: a cross-sectional study using 1-year loyalty card data
Source: Am J Clin Nutr. 2024 Mar 7;119(5):1346–53. doi: 10.1016/j.ajcnut.2024.03.003 (PMC11130695; doi:10.1016/j.ajcnut.2024.03.003)
Supplement: Multimedia component 1 [file mmc1.docx]

Paper title: The dynamics in food selection stemming from price awareness and perceived income adequacy: a cross-sectional study using 1-year loyalty-card data

Main author: Mikael Fogelholm

SUPPLEMENTARY MATERIAL

**Contents:**

**Supplemental Table 1.** Distribution of expenditure (%) and energy (%) across all food groups and level of Perceived Income Adequacy (PIA): 1) I/we 1; 2) I/we have to compromise on purchases from time to time; 3) I/we manage when I/we purchase carefully; 4) I/we 4; 5) I/we 5.

**Supplemental Figure 1.** The flow chart of participant recruitment, inclusions, and exclusions. PIA = Perceived Income Adequacy. The recruitment, including an analysis of potential bias in terms of population representativeness, has been published by Vuorinen et al. [ref. 20 in the main paper].

**Supplemental Figure 2.** The association between perceived income adequacy (PIA) (5-level grouping variable), and median price per energy (euro/MJ) and proportion of energy (%) (simultaneous outcome variables) in different food groups. The arrow indicates to which direction the bivariate outcome changes following increase in PIA (1🡪5), except for alcohol beverages, in which PIA levels 4 and 5 are in reverse order (indicated by reverse arrow). Significant trend (p<0.001), with Jonckheere-Terpstra test with false discovery rate correction and IPW indicated by: a=price per MJ, b=proportion of expenditure

**Supplemental Figure 3** The association between perceived income adequacy (PIA) (5-level grouping variable), and median price per kg and per MJ (simultaneous outcome variables) in different food groups. All correlations were statistically significant (p<0.001).

**Supplemental Figure 4.** The association between perceived income adequacy (PIA) (5-level grouping variable), and median price per energy (euro/MJ) and Nutrient Rich Food Index (NRFI; a measure of nutritional profile) (simultaneous outcome variables) in different food groups. Significant trend (p<0.001) with Jonckheere-Terpstra test with false discovery rate correction and IPW. a=price per kg; b=median NRFI. Only food groups with both a and b significant are indicated.

**Supplemental Table 1.** Distribution of expenditure (%) and energy (%) across all food groups and level of Perceived Income Adequacy (PIA): 1) I/we 1; 2) I/we have to compromise on purchases from time to time; 3) I/we manage when I/we purchase carefully; 4) I/we 4; 5) I/we 5.

|  |  |  | Expenditure, % | | |  | Energy, % | | |
| --- | --- | --- | --- | --- | --- | --- | --- | --- | --- |
| Food group | Perceived Income Adequacy level |  | Quartile1 | Median | Quartile 3 |  | Quartile 1 | Median | Quartile 3 |
| Alcoholic beverages | 1 |  | 0.5 | 2.9 | 9.6 |  | 0.1 | 0.8 | 3.1 |
| Alcoholic beverages | 2 |  | 0.5 | 2.3 | 6.6 |  | 0.1 | 0.6 | 2 |
| Alcoholic beverages | 3 |  | 0.4 | 2.1 | 6.4 |  | 0.1 | 0.6 | 2 |
| Alcoholic beverages | 4 |  | 0.5 | 2.4 | 6.4 |  | 0.1 | 0.7 | 2.1 |
| Alcoholic beverages | 5 |  | 0.4 | 2.3 | 6.6 |  | 0.1 | 0.7 | 2.2 |
| Alcoholic beverages | Overall |  | 0.4 | 2.3 | 6.6 |  | 0.1 | 0.6 | 2.1 |
| Baby foods | 1 |  | 0 | 0 | 0.1 |  | 0 | 0 | 0.1 |
| Baby foods | 2 |  | 0 | 0 | 0.2 |  | 0 | 0 | 0.1 |
| Baby foods | 3 |  | 0 | 0 | 0.2 |  | 0 | 0 | 0.1 |
| Baby foods | 4 |  | 0 | 0 | 0.1 |  | 0 | 0 | 0 |
| Baby foods | 5 |  | 0 | 0 | 0.1 |  | 0 | 0 | 0 |
| Baby foods | Overall |  | 0 | 0 | 0.1 |  | 0 | 0 | 0.1 |
| Bakery | 1 |  | 3.3 | 5.5 | 7.9 |  | 6 | 9 | 12.6 |
| Bakery | 2 |  | 3.7 | 5.5 | 8 |  | 6.6 | 9.4 | 12.9 |
| Bakery | 3 |  | 3.5 | 5.4 | 7.8 |  | 6.5 | 9.4 | 12.8 |
| Bakery | 4 |  | 3.2 | 5 | 7.4 |  | 6.2 | 9 | 12.6 |
| Bakery | 5 |  | 2.9 | 4.7 | 7.1 |  | 5.9 | 8.8 | 12.4 |
| Bakery | Overall |  | 3.3 | 5.1 | 7.6 |  | 6.3 | 9.1 | 12.7 |
| Baking products | 1 |  | 0 | 0.1 | 0.3 |  | 0 | 0 | 0.2 |
| Baking products | 2 |  | 0 | 0.2 | 0.4 |  | 0 | 0.1 | 0.2 |
| Baking products | 3 |  | 0 | 0.1 | 0.4 |  | 0 | 0.1 | 0.2 |
| Baking products | 4 |  | 0 | 0.1 | 0.3 |  | 0 | 0.1 | 0.2 |
| Baking products | 5 |  | 0 | 0.1 | 0.3 |  | 0 | 0.1 | 0.2 |
| Baking products | Overall |  | 0 | 0.1 | 0.3 |  | 0 | 0.1 | 0.2 |
| Bottled water | 1 |  | 0.1 | 0.4 | 1.1 |  | 0 | 0 | 0 |
| Bottled water | 2 |  | 0.2 | 0.5 | 1.2 |  | 0 | 0 | 0 |
| Bottled water | 3 |  | 0.1 | 0.5 | 1.2 |  | 0 | 0 | 0 |
| Bottled water | 4 |  | 0.1 | 0.5 | 1.2 |  | 0 | 0 | 0 |
| Bottled water | 5 |  | 0.1 | 0.4 | 1.1 |  | 0 | 0 | 0 |
| Bottled water | Overall |  | 0.1 | 0.5 | 1.2 |  | 0 | 0 | 0 |
| Bread | 1 |  | 2.3 | 3.9 | 5.7 |  | 5.2 | 7.8 | 10.8 |
| Bread | 2 |  | 2.8 | 4.3 | 6.1 |  | 6.1 | 8.6 | 11.4 |
| Bread | 3 |  | 3.2 | 4.8 | 6.7 |  | 6.6 | 9.1 | 12.2 |
| Bread | 4 |  | 3.5 | 5 | 7 |  | 7.1 | 9.7 | 12.8 |
| Bread | 5 |  | 3.6 | 5.3 | 7.4 |  | 7.5 | 10.3 | 13.8 |
| Bread | Overall |  | 3.3 | 4.8 | 6.8 |  | 6.7 | 9.4 | 12.5 |
| Cheeses | 1 |  | 3.2 | 4.9 | 7.4 |  | 3.9 | 5.9 | 8.9 |
| Cheeses | 2 |  | 3.7 | 5.5 | 7.8 |  | 4.1 | 6.2 | 8.9 |
| Cheeses | 3 |  | 3.9 | 5.9 | 8.1 |  | 4.2 | 6.4 | 8.9 |
| Cheeses | 4 |  | 4.4 | 6.4 | 8.6 |  | 4.6 | 6.8 | 9.3 |
| Cheeses | 5 |  | 4.6 | 6.7 | 9.2 |  | 4.7 | 6.8 | 9.6 |
| Cheeses | Overall |  | 4.1 | 6.1 | 8.4 |  | 4.4 | 6.6 | 9.1 |
| Chewing gum | 1 |  | 0 | 0 | 0.3 |  | 0 | 0 | 0.1 |
| Chewing gum | 2 |  | 0 | 0.1 | 0.4 |  | 0 | 0 | 0.1 |
| Chewing gum | 3 |  | 0 | 0.1 | 0.4 |  | 0 | 0 | 0.1 |
| Chewing gum | 4 |  | 0 | 0.1 | 0.4 |  | 0 | 0 | 0.1 |
| Chewing gum | 5 |  | 0 | 0 | 0.4 |  | 0 | 0 | 0.1 |
| Chewing gum | Overall |  | 0 | 0.1 | 0.4 |  | 0 | 0 | 0.1 |
| Cocoa | 1 |  | 0 | 0 | 0.2 |  | 0 | 0 | 0.3 |
| Cocoa | 2 |  | 0 | 0 | 0.2 |  | 0 | 0 | 0.3 |
| Cocoa | 3 |  | 0 | 0 | 0.2 |  | 0 | 0 | 0.3 |
| Cocoa | 4 |  | 0 | 0 | 0.1 |  | 0 | 0 | 0.2 |
| Cocoa | 5 |  | 0 | 0 | 0.1 |  | 0 | 0 | 0.1 |
| Cocoa | Overall |  | 0 | 0 | 0.1 |  | 0 | 0 | 0.2 |
| Coffee | 1 |  | 0.4 | 1.2 | 2.6 |  | 0.1 | 0.3 | 0.7 |
| Coffee | 2 |  | 0.5 | 1.3 | 2.5 |  | 0.1 | 0.4 | 0.7 |
| Coffee | 3 |  | 0.5 | 1.4 | 2.6 |  | 0.1 | 0.4 | 0.7 |
| Coffee | 4 |  | 0.6 | 1.5 | 2.7 |  | 0.2 | 0.4 | 0.8 |
| Coffee | 5 |  | 0.6 | 1.5 | 2.7 |  | 0.2 | 0.4 | 0.8 |
| Coffee | Overall |  | 0.5 | 1.4 | 2.6 |  | 0.1 | 0.4 | 0.8 |
| Desserts | 1 |  | 0.1 | 0.3 | 0.8 |  | 0 | 0.2 | 0.5 |
| Desserts | 2 |  | 0.1 | 0.4 | 0.8 |  | 0.1 | 0.3 | 0.6 |
| Desserts | 3 |  | 0.1 | 0.4 | 0.8 |  | 0.1 | 0.3 | 0.7 |
| Desserts | 4 |  | 0.1 | 0.4 | 0.8 |  | 0.1 | 0.3 | 0.7 |
| Desserts | 5 |  | 0.1 | 0.3 | 0.7 |  | 0.1 | 0.3 | 0.7 |
| Desserts | Overall |  | 0.1 | 0.4 | 0.8 |  | 0.1 | 0.3 | 0.7 |
| Dietary suppleme | 1 |  | 0 | 0.1 | 0.7 |  | . | . | . |
| Dietary suppleme | 2 |  | 0 | 0.1 | 0.7 |  | . | . | . |
| Dietary suppleme | 3 |  | 0 | 0.1 | 0.7 |  | . | . | . |
| Dietary suppleme | 4 |  | 0 | 0 | 0.6 |  | . | . | . |
| Dietary suppleme | 5 |  | 0 | 0 | 0.6 |  | . | . | . |
| Dietary suppleme | Overall |  | 0 | 0 | 0.6 |  | . | . | . |
| Dried fruit nut | 1 |  | 0 | 0.2 | 0.8 |  | 0 | 0.3 | 0.9 |
| Dried fruit & nut | 2 |  | 0.1 | 0.4 | 1 |  | 0.1 | 0.4 | 1.1 |
| Dried fruit & nut | 3 |  | 0.1 | 0.5 | 1.3 |  | 0.1 | 0.5 | 1.5 |
| Dried fruit & nut | 4 |  | 0.2 | 0.6 | 1.4 |  | 0.2 | 0.7 | 1.8 |
| Dried fruit & nut | 5 |  | 0.2 | 0.8 | 1.8 |  | 0.3 | 0.9 | 2.3 |
| Dried fruit & nut | Overall |  | 0.1 | 0.5 | 1.3 |  | 0.1 | 0.6 | 1.6 |
| Edible fat | 1 |  | 1 | 1.8 | 2.8 |  | 5.1 | 7.7 | 10.7 |
| Edible fat | 2 |  | 1.2 | 1.9 | 2.8 |  | 5.4 | 7.8 | 10.6 |
| Edible fat | 3 |  | 1.3 | 2.1 | 3.1 |  | 5.7 | 8.2 | 11.1 |
| Edible fat | 4 |  | 1.3 | 2 | 3 |  | 5.5 | 8.2 | 11.1 |
| Edible fat | 5 |  | 1.2 | 2 | 3.1 |  | 5.4 | 8.1 | 11.1 |
| Edible fat | Overall |  | 1.3 | 2 | 3 |  | 5.5 | 8.1 | 11 |
| Eggs | 1 |  | 0.4 | 0.7 | 1.2 |  | 0.5 | 1 | 1.7 |
| Eggs | 2 |  | 0.4 | 0.8 | 1.3 |  | 0.6 | 1 | 1.7 |
| Eggs | 3 |  | 0.4 | 0.8 | 1.4 |  | 0.6 | 1.1 | 1.8 |
| Eggs | 4 |  | 0.4 | 0.8 | 1.4 |  | 0.6 | 1.1 | 1.8 |
| Eggs | 5 |  | 0.4 | 0.8 | 1.4 |  | 0.6 | 1.1 | 1.7 |
| Eggs | Overall |  | 0.4 | 0.8 | 1.3 |  | 0.6 | 1.1 | 1.8 |
| Fish & seafood | 1 |  | 0.7 | 1.8 | 3.6 |  | 0.4 | 0.9 | 1.7 |
| Fish & seafood | 2 |  | 1 | 2.2 | 4 |  | 0.5 | 1 | 1.7 |
| Fish & seafood | 3 |  | 1.3 | 2.8 | 4.9 |  | 0.6 | 1.2 | 2.1 |
| Fish & seafood | 4 |  | 1.6 | 3.4 | 5.9 |  | 0.7 | 1.4 | 2.4 |
| Fish & seafood | 5 |  | 2.2 | 4.4 | 7.3 |  | 0.8 | 1.7 | 2.9 |
| Fish & seafood | Overall |  | 1.4 | 3 | 5.4 |  | 0.6 | 1.3 | 2.2 |
| Fruit & berries | 1 |  | 1.7 | 3.4 | 6.5 |  | 1 | 2.1 | 3.9 |
| Fruit & berries | 2 |  | 2.4 | 4.5 | 7 |  | 1.4 | 2.6 | 4.3 |
| Fruit & berries | 3 |  | 2.9 | 5 | 7.8 |  | 1.7 | 3 | 4.9 |
| Fruit & berries | 4 |  | 3.2 | 5.4 | 8.2 |  | 2 | 3.4 | 5.3 |
| Fruit & berries | 5 |  | 3.4 | 5.8 | 9 |  | 2.2 | 3.7 | 5.8 |
| Fruit & berries | Overall |  | 2.9 | 5.1 | 8 |  | 1.8 | 3.1 | 5.1 |
| Fruit juice | 1 |  | 0.1 | 0.3 | 0.8 |  | 0 | 0.2 | 0.7 |
| Fruit juice | 2 |  | 0.1 | 0.4 | 1.1 |  | 0.1 | 0.3 | 0.8 |
| Fruit juice | 3 |  | 0.1 | 0.4 | 1.1 |  | 0.1 | 0.3 | 0.8 |
| Fruit juice | 4 |  | 0.1 | 0.5 | 1.3 |  | 0.1 | 0.3 | 1 |
| Fruit juice | 5 |  | 0.1 | 0.5 | 1.5 |  | 0.1 | 0.4 | 1.2 |
| Fruit juice | Overall |  | 0.1 | 0.4 | 1.2 |  | 0.1 | 0.3 | 0.9 |
| Grain-based food | 1 |  | 0.8 | 1.6 | 2.7 |  | 3.3 | 5.5 | 8.3 |
| Grain-based food | 2 |  | 1 | 1.8 | 2.8 |  | 3.6 | 5.8 | 8.7 |
| Grain-based food | 3 |  | 1 | 1.8 | 2.9 |  | 3.6 | 6.1 | 9.1 |
| Grain-based food | 4 |  | 1 | 1.7 | 2.8 |  | 3.6 | 5.9 | 9 |
| Grain-based food | 5 |  | 0.9 | 1.7 | 2.8 |  | 3.4 | 6 | 9.1 |
| Grain-based food | Overall |  | 1 | 1.8 | 2.8 |  | 3.6 | 5.9 | 9 |
| Jam & marmalade | 1 |  | 0 | 0.2 | 0.4 |  | 0 | 0.2 | 0.5 |
| Jam & marmalade | 2 |  | 0 | 0.2 | 0.5 |  | 0.1 | 0.3 | 0.6 |
| Jam & marmalade | 3 |  | 0 | 0.2 | 0.5 |  | 0.1 | 0.2 | 0.5 |
| Jam & marmalade | 4 |  | 0 | 0.2 | 0.5 |  | 0.1 | 0.2 | 0.5 |
| Jam & marmalade | 5 |  | 0 | 0.2 | 0.5 |  | 0 | 0.2 | 0.5 |
| Jam & marmalade | Overall |  | 0 | 0.2 | 0.5 |  | 0 | 0.2 | 0.5 |
| Low-sugar beverages | 1 |  | 0.1 | 0.5 | 1.6 |  | 0 | 0 | 0.3 |
| Low-sugar beverages | 2 |  | 0.1 | 0.5 | 1.6 |  | 0 | 0 | 0.3 |
| Low-sugar beverages | Low-sugar beverages |  | 0.1 | 0.4 | 1.4 |  | 0 | 0 | 0.2 |
| Low-sugar beverages | 4 |  | 0 | 0.3 | 1.3 |  | 0 | 0 | 0.1 |
| Low-sugar beverages | 5 |  | 0 | 0.3 | 1.2 |  | 0 | 0 | 0.1 |
| Low-sugar beverages | Overall |  | 0.1 | 0.4 | 1.4 |  | 0 | 0 | 0.2 |
| Mayonnaise & salad | 1 |  | 0.1 | 0.5 | 1.2 |  | 0.1 | 0.4 | 0.9 |
| Mayonnaise & salad | 2 |  | 0.2 | 0.6 | 1.4 |  | 0.1 | 0.4 | 1 |
| Mayonnaise & salad | 3 |  | 0.1 | 0.5 | 1.4 |  | 0.1 | 0.4 | 1 |
| Mayonnaise & salad | 4 |  | 0.1 | 0.5 | 1.4 |  | 0.1 | 0.4 | 1 |
| Mayonnaise & salad | 5 |  | 0.1 | 0.5 | 1.3 |  | 0.1 | 0.3 | 1 |
| Mayonnaise & salad | Overall |  | 0.1 | 0.5 | 1.4 |  | 0.1 | 0.4 | 1 |
| Meal ingredients | 1 |  | 0.1 | 0.4 | 0.8 |  | 0 | 0.3 | 0.8 |
| Meal ingredients | 2 |  | 0.1 | 0.4 | 0.8 |  | 0.1 | 0.4 | 0.9 |
| Meal ingredients | 3 |  | 0.1 | 0.4 | 0.8 |  | 0.1 | 0.3 | 0.8 |
| Meal ingredients | 4 |  | 0.1 | 0.3 | 0.7 |  | 0 | 0.3 | 0.8 |
| Meal ingredients | 5 |  | 0 | 0.3 | 0.7 |  | 0 | 0.3 | 0.7 |
| Meal ingredients | Overall |  | 0.1 | 0.4 | 0.7 |  | 0 | 0.3 | 0.8 |
| Milk & dairy prod | 1 |  | 6 | 9.6 | 13.9 |  | 7.4 | 11.1 | 14.9 |
| Milk & dairy prod | 2 |  | 7.2 | 10.7 | 14.5 |  | 8.3 | 11.6 | 15.1 |
| Milk & dairy prod | 3 |  | 7.6 | 11 | 14.7 |  | 8.6 | 12 | 15.4 |
| Milk & dairy prod | 4 |  | 7.4 | 10.7 | 14.3 |  | 8.6 | 11.8 | 15.2 |
| Milk & dairy prod | 5 |  | 7.2 | 10.5 | 14.3 |  | 8.4 | 11.8 | 15.2 |
| Milk & dairy prod | Overall |  | 7.4 | 10.7 | 14.4 |  | 8.5 | 11.8 | 15.2 |
| Miscellaneous | 1 |  | 0.1 | 0.5 | 1.2 |  | . | . | . |
| Miscellaneous | 2 |  | 0.2 | 0.6 | 1.3 |  | . | . | . |
| Miscellaneous | 3 |  | 0.1 | 0.5 | 1.3 |  | . | . | . |
| Miscellaneous | 4 |  | 0.2 | 0.6 | 1.3 |  | . | . | . |
| Miscellaneous | 5 |  | 0.1 | 0.6 | 1.3 |  | . | . | . |
| Miscellaneous | Overall |  | 0.2 | 0.5 | 1.3 |  | . | . | . |
| Non-alcoholic beverages | 1 |  | 0 | 0 | 0 |  | 0 | 0 | 0 |
| Non-alcoholic beverages | 2 |  | 0 | 0 | 0 |  | 0 | 0 | 0 |
| Non-alcoholic beverages | 3 |  | 0 | 0 | 0 |  | 0 | 0 | 0 |
| Non-alcoholic beverages | 4 |  | 0 | 0 | 0.1 |  | 0 | 0 | 0 |
| Non-alcoholic beverages | 5 |  | 0 | 0 | 0.1 |  | 0 | 0 | 0 |
| Non-alcoholic beverages | Overall |  | 0 | 0 | 0 |  | 0 | 0 | 0 |
| Plant based products | 1 |  | 0 | 0.1 | 0.7 |  | 0 | 0.1 | 0.6 |
| Plant based products | 2 |  | 0 | 0.2 | 1.1 |  | 0 | 0.1 | 0.8 |
| Plant based products | 3 |  | 0 | 0.2 | 1.1 |  | 0 | 0.1 | 0.8 |
| Plant based products | 4 |  | 0 | 0.2 | 1.1 |  | 0 | 0.2 | 0.9 |
| Plant based products | 5 |  | 0 | 0.2 | 1.1 |  | 0 | 0.2 | 0.9 |
| Plant based products | Overall |  | 0 | 0.2 | 1.1 |  | 0 | 0.2 | 0.9 |
| Plant protein | 1 |  | 0 | 0.1 | 0.5 |  | 0 | 0.1 | 0.5 |
| Plant protein | 2 |  | 0 | 0.2 | 0.6 |  | 0 | 0.2 | 0.5 |
| Plant protein | 3 |  | 0 | 0.2 | 0.6 |  | 0 | 0.2 | 0.5 |
| Plant protein | 4 |  | 0 | 0.2 | 0.7 |  | 0 | 0.2 | 0.6 |
| Plant protein | 5 |  | 0 | 0.2 | 0.7 |  | 0 | 0.2 | 0.6 |
| Plant protein | Overall |  | 0 | 0.2 | 0.6 |  | 0 | 0.2 | 0.6 |
| Potatoes | 1 |  | 0.5 | 1 | 1.6 |  | 1.3 | 2.4 | 3.7 |
| Potatoes | 2 |  | 0.6 | 1 | 1.6 |  | 1.3 | 2.3 | 3.6 |
| Potatoes | 3 |  | 0.6 | 1 | 1.6 |  | 1.3 | 2.3 | 3.4 |
| Potatoes | 4 |  | 0.5 | 0.9 | 1.4 |  | 1.2 | 2.1 | 3.3 |
| Potatoes | 5 |  | 0.4 | 0.8 | 1.3 |  | 1.1 | 2 | 3.1 |
| Potatoes | Overall |  | 0.5 | 0.9 | 1.5 |  | 1.2 | 2.2 | 3.3 |
| Poultry and dish | 1 |  | 0.9 | 2.3 | 4 |  | 0.7 | 1.5 | 2.6 |
| Poultry and dish | 2 |  | 1.2 | 2.7 | 4.5 |  | 0.8 | 1.6 | 2.8 |
| Poultry and dish | 3 |  | 1.4 | 2.9 | 4.5 |  | 0.8 | 1.6 | 2.7 |
| Poultry and dish | 4 |  | 1.4 | 2.9 | 4.6 |  | 0.8 | 1.7 | 2.7 |
| Poultry and dish | 5 |  | 1.2 | 2.7 | 4.5 |  | 0.7 | 1.6 | 2.6 |
| Poultry and dish | Overall |  | 1.3 | 2.8 | 4.5 |  | 0.8 | 1.6 | 2.7 |
| Red & processed meat | 1 |  | 6.7 | 10.1 | 13.9 |  | 6.1 | 9.8 | 13.9 |
| Red & processed meat | 2 |  | 6.8 | 10.3 | 13.9 |  | 5.7 | 9.1 | 12.8 |
| Red & processed meat | 3 |  | 7.1 | 10.9 | 14.8 |  | 5.6 | 8.9 | 12.4 |
| Red & processed meat | 4 |  | 7.2 | 10.8 | 14.6 |  | 5.3 | 8.3 | 11.7 |
| Red & processed meat | 5 |  | 6.5 | 10.3 | 14.3 |  | 4.6 | 7.4 | 10.7 |
| Red & processed meat | Overall |  | 7 | 10.7 | 14.5 |  | 5.4 | 8.5 | 12 |
| Sauces | 1 |  | 0.2 | 0.5 | 1 |  | 0.1 | 0.6 | 1.4 |
| Sauces | 2 |  | 0.2 | 0.6 | 1.1 |  | 0.2 | 0.7 | 1.4 |
| Sauces | 3 |  | 0.2 | 0.5 | 1 |  | 0.2 | 0.6 | 1.3 |
| Sauces | 4 |  | 0.2 | 0.5 | 1 |  | 0.2 | 0.6 | 1.3 |
| Sauces | 5 |  | 0.2 | 0.5 | 0.9 |  | 0.1 | 0.5 | 1.1 |
| Sauces | Overall |  | 0.2 | 0.5 | 1 |  | 0.2 | 0.6 | 1.3 |
| Seasoning | 1 |  | 0.6 | 1 | 1.5 |  | 0.3 | 0.5 | 0.7 |
| Seasoning | 2 |  | 0.6 | 1 | 1.5 |  | 0.3 | 0.5 | 0.7 |
| Seasoning | 3 |  | 0.6 | 1 | 1.4 |  | 0.3 | 0.4 | 0.7 |
| Seasoning | 4 |  | 0.6 | 0.9 | 1.4 |  | 0.2 | 0.4 | 0.7 |
| Seasoning | 5 |  | 0.5 | 0.8 | 1.3 |  | 0.2 | 0.4 | 0.6 |
| Seasoning | Overall |  | 0.6 | 0.9 | 1.4 |  | 0.2 | 0.4 | 0.7 |
| Snacks | 1 |  | 0.4 | 0.9 | 1.9 |  | 0.7 | 1.9 | 3.9 |
| Snacks | 2 |  | 0.4 | 1 | 1.9 |  | 0.8 | 2 | 3.7 |
| Snacks | 3 |  | 0.2 | 0.8 | 1.6 |  | 0.4 | 1.5 | 3.1 |
| Snacks | 4 |  | 0.2 | 0.6 | 1.4 |  | 0.3 | 1.3 | 2.8 |
| Snacks | 5 |  | 0.1 | 0.5 | 1.1 |  | 0.2 | 0.9 | 2.3 |
| Snacks | Overall |  | 0.2 | 0.7 | 1.5 |  | 0.4 | 1.4 | 3 |
| Sugarsweetened beverages | 1 |  | 0.7 | 1.8 | 3.6 |  | 0.6 | 1.7 | 3.7 |
| Sugarsweetened beverages | 2 |  | 0.7 | 1.7 | 3.2 |  | 0.6 | 1.5 | 3.1 |
| Sugarsweetened beverages | 3 |  | 0.5 | 1.3 | 2.7 |  | 0.4 | 1.1 | 2.5 |
| Sugarsweetened beverages | 4 |  | 0.5 | 1.2 | 2.4 |  | 0.4 | 1 | 2.2 |
| Sugarsweetened beverages | 5 |  | 0.4 | 0.9 | 2 |  | 0.3 | 0.8 | 1.9 |
| Sugarsweetened beverages | Overall |  | 0.5 | 1.3 | 2.6 |  | 0.4 | 1.1 | 2.4 |
| Sweeteners | 1 |  | 0.1 | 0.2 | 0.5 |  | 0.4 | 1.3 | 2.9 |
| Sweeteners | 2 |  | 0.1 | 0.2 | 0.5 |  | 0.4 | 1.4 | 2.8 |
| Sweeteners | 3 |  | 0.1 | 0.3 | 0.6 |  | 0.5 | 1.5 | 3 |
| Sweeteners | 4 |  | 0.1 | 0.2 | 0.5 |  | 0.4 | 1.4 | 2.8 |
| Sweeteners | 5 |  | 0.1 | 0.2 | 0.6 |  | 0.4 | 1.3 | 2.8 |
| Sweeteners | Overall |  | 0.1 | 0.2 | 0.5 |  | 0.4 | 1.4 | 2.9 |
| Sweets & chocolate | 1 |  | 2 | 3.8 | 6.3 |  | 2.3 | 4.5 | 7.6 |
| Sweets & chocolate | 2 |  | 2.3 | 4 | 6.4 |  | 2.7 | 4.8 | 7.9 |
| Sweets & chocolate | 3 |  | 1.8 | 3.3 | 5.4 |  | 2 | 3.9 | 6.7 |
| Sweets & chocolate | 4 |  | 1.6 | 3 | 5 |  | 1.9 | 3.7 | 6.3 |
| Sweets & chocolate | 5 |  | 1.3 | 2.6 | 4.5 |  | 1.6 | 3.2 | 5.8 |
| Sweets & chocolate | Overall |  | 1.7 | 3.2 | 5.3 |  | 2 | 3.9 | 6.6 |
| Tea | 1 |  | 0 | 0 | 0.3 |  | 0 | 0 | 0 |
| Tea | 2 |  | 0 | 0.1 | 0.3 |  | 0 | 0 | 0 |
| Tea | 3 |  | 0 | 0.1 | 0.3 |  | 0 | 0 | 0 |
| Tea | 4 |  | 0 | 0.1 | 0.3 |  | 0 | 0 | 0 |
| Tea | 5 |  | 0 | 0.1 | 0.4 |  | 0 | 0 | 0 |
| Tea | Overall |  | 0 | 0.1 | 0.3 |  | 0 | 0 | 0 |
| Tobacco products | 1 |  | 0 | 0.3 | 10.4 |  | . | . | . |
| Tobacco products | 2 |  | 0 | 0 | 4.3 |  | . | . | . |
| Tobacco products | 3 |  | 0 | 0 | 0.7 |  | . | . | . |
| Tobacco products | 4 |  | 0 | 0 | 0 |  | . | . | . |
| Tobacco products | 5 |  | 0 | 0 | 0 |  | . | . | . |
| Tobacco products | Overall |  | 0 | 0 | 0.5 |  | . | . | . |
| Uncategorized | 1 |  | 0 | 0 | 0 |  | 0 | 0 | 0.1 |
| Uncategorized | 2 |  | 0 | 0 | 0 |  | 0 | 0 | 0.1 |
| Uncategorized | 3 |  | 0 | 0 | 0 |  | 0 | 0 | 0.1 |
| Uncategorized | 4 |  | 0 | 0 | 0 |  | 0 | 0 | 0 |
| Uncategorized | 5 |  | 0 | 0 | 0 |  | 0 | 0 | 0 |
| Uncategorized | Overall |  | 0 | 0 | 0 |  | 0 | 0 | 0.1 |
| Vegetables | 1 |  | 3.3 | 5.6 | 8.8 |  | 0.9 | 1.6 | 2.5 |
| Vegetables | 2 |  | 4.3 | 6.7 | 9.7 |  | 1.1 | 1.8 | 2.7 |
| Vegetables | 3 |  | 5 | 7.6 | 10.7 |  | 1.3 | 2 | 2.9 |
| Vegetables | 4 |  | 5.6 | 8.2 | 11.2 |  | 1.4 | 2.2 | 3.1 |
| Vegetables | 5 |  | 6.1 | 8.9 | 11.9 |  | 1.6 | 2.4 | 3.3 |
| Vegetables | Overall |  | 5.1 | 7.8 | 10.9 |  | 1.3 | 2.1 | 3 |
|  |  |  |  |  |  |  |  |  |  |


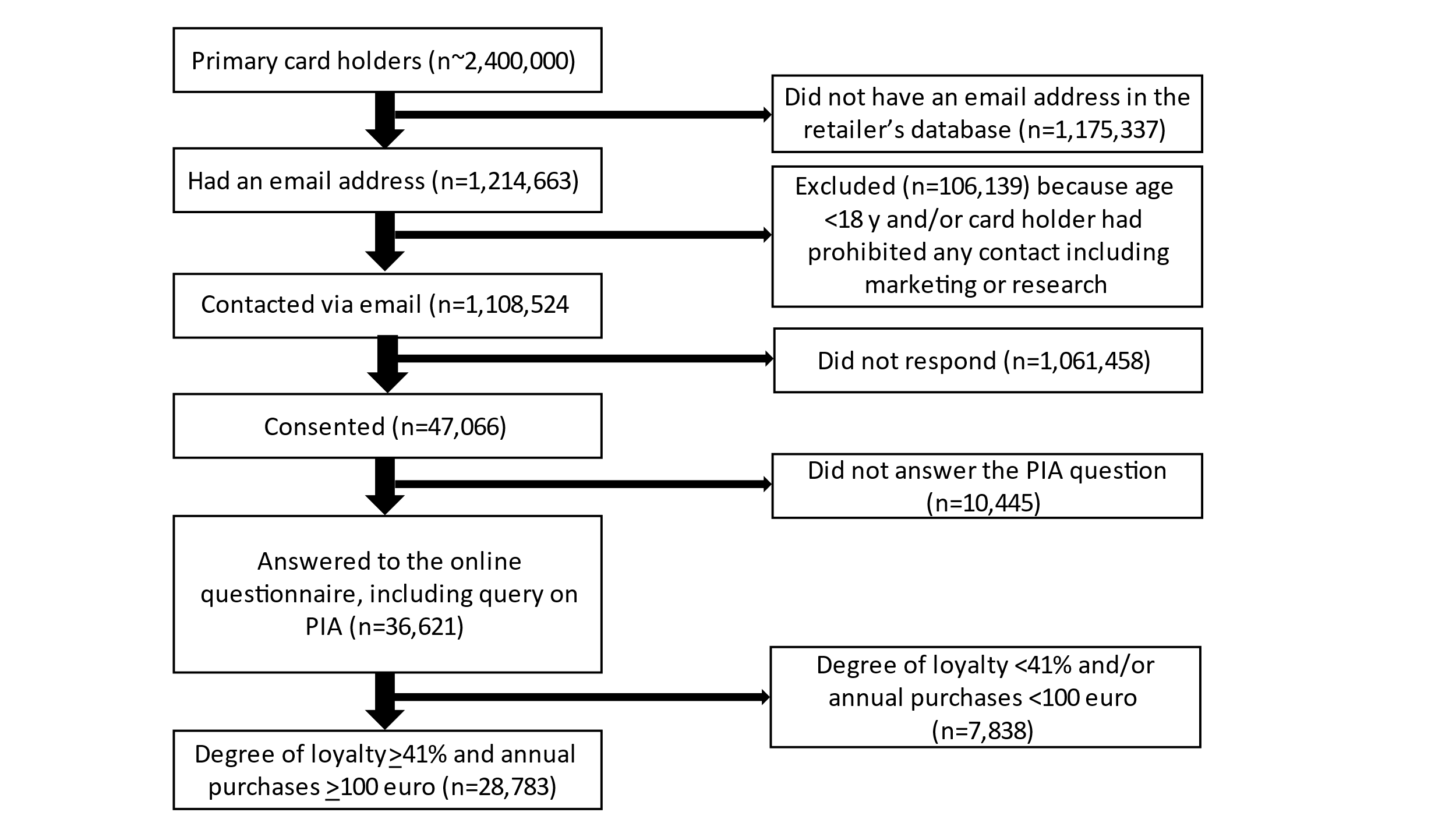


**Supplemental Figure 1.** The flow chart of participant recruitment, inclusions, and exclusions. PIA = Perceived Income Adequacy. The recruitment, including an analysis of potential bias in terms of population representativeness, has been published by Vuorinen et al. [ref. 20 in the main paper].


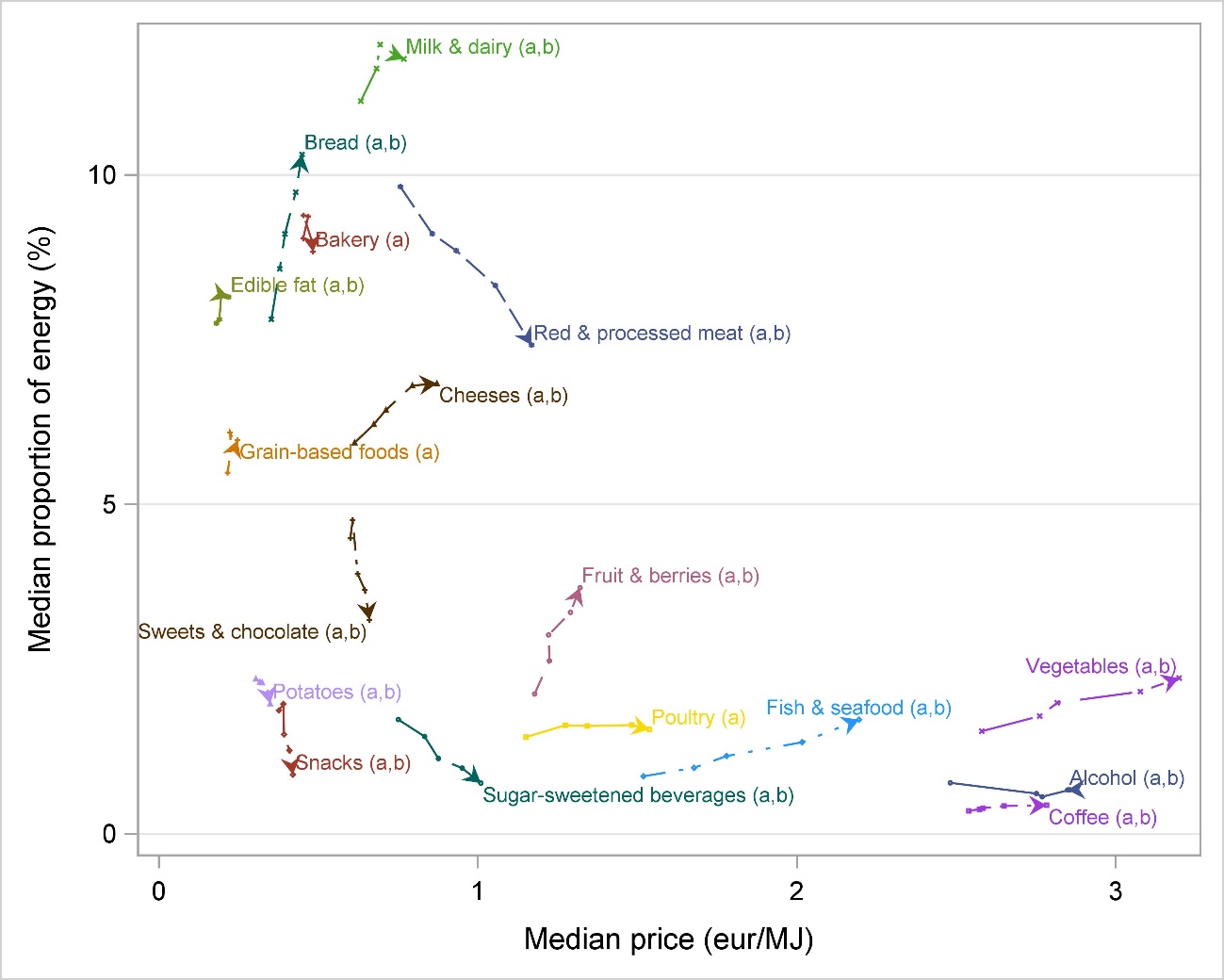


**Supplemental Figure 2.** The association between perceived income adequacy (PIA) (5-level grouping variable), and median price per energy (euro/MJ) and proportion of energy (%) (simultaneous outcome variables) in different food groups. The arrow indicates to which direction the bivariate outcome changes following increase in PIA (1🡪5), except for alcohol beverages, in which PIA levels 4 and 5 are in reverse order (indicated by reverse arrow). Significant trend (p<0.001), with Jonckheere-Terpstra test with false discovery rate correction and IPW indicated by: a=price per MJ, b=proportion of expenditure


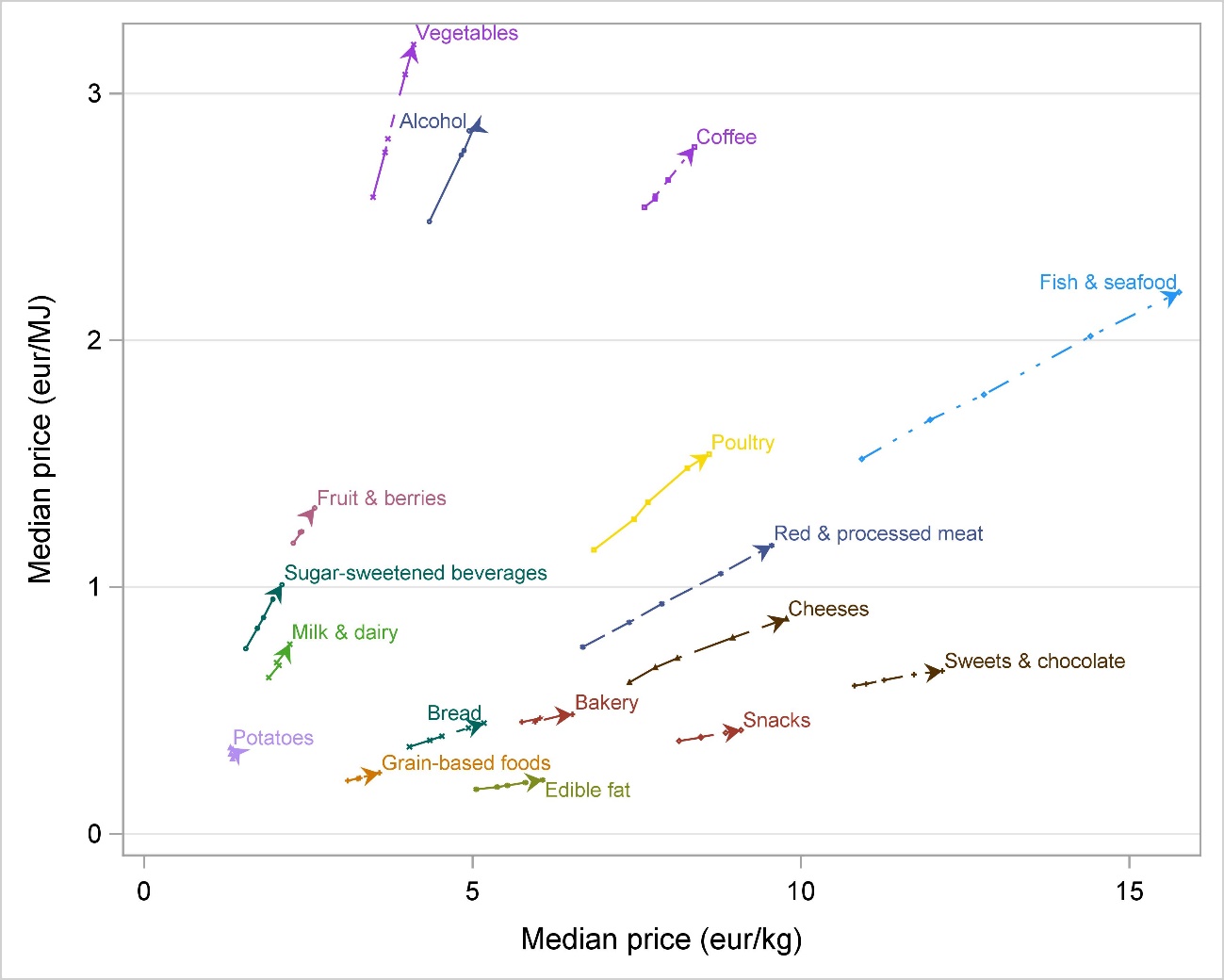


**Supplemental Figure 3** The association between perceived income adequacy (PIA) (5-level grouping variable), and median price per kg and per MJ (simultaneous outcome variables) in different food groups. All correlations were statistically significant (p<0.001).


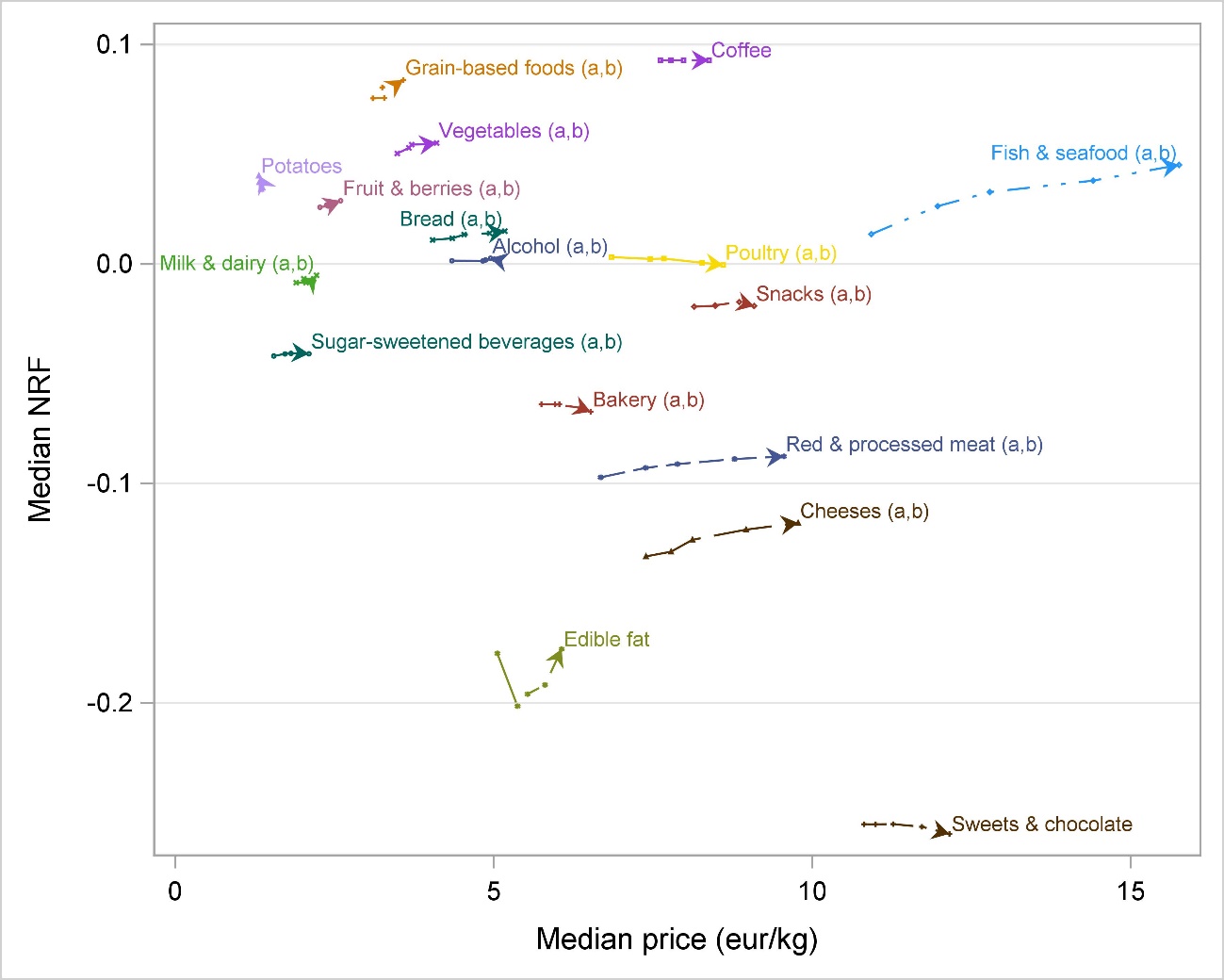


**Supplemental Figure 4.** The association between perceived income adequacy (PIA) (5-level grouping variable), and median price (euro) per kg and Nutrient Rich Food Index (NRFI; a measure of nutritional profile) (simultaneous outcome variables) in different food groups. Significant trend (p<0.001) with Jonckheere-Terpstra test with false discovery rate correction and IPW. a=price per kg; b=median NRFI. Only food groups with both a and b significant are indicated.
